# Pathogenic Role of Cytokines in Rheumatoid Arthritis

**DOI:** 10.3390/jcm14186409

**Published:** 2025-09-11

**Authors:** Sho Fujimoto, Hiroaki Niiro

**Affiliations:** Department of Medical Education, Faculty of Medical Sciences, Kyushu University, Fukuoka 812-8582, Japan; fujimoto.sho.366@m.kyushu-u.ac.jp

**Keywords:** rheumatoid arthritis, cytokines, autoimmunity, synovitis, joint destruction

## Abstract

Rheumatoid arthritis (RA) is a systemic autoimmune disease characterized by a multistep pathogenesis, from the preclinical phase of autoantibody emergence to the clinical onset of synovitis and joint destruction. Cytokines play central roles throughout this progression by orchestrating immune cell activation, tissue inflammation, and bone erosion. In the preclinical phase, several cytokines, including IL-12, IL-6, IL-21 and TGF-β, promote Tfh and Tph cell differentiation, helping autoreactive B cells to produce ACPA. During the clinical phase, TNF-α, IL-6, and IL-1β drive synovitis by activating macrophages and fibroblast-like synoviocytes, while also promoting RANKL (Receptor Activator of Nuclear factor κB Ligand) expression and osteoclast differentiation. This review highlights the pathogenic role of cytokines in RA and discusses their relevance as biomarkers and therapeutic targets. A better understanding of cytokine networks may offer new opportunities for early intervention and disease prevention in RA.

## 1. Introduction

Rheumatoid arthritis (RA) is an autoimmune disease characterized by chronic synovial inflammation and progressive bone destruction and resulting in severe joint deformity and functional impairment. The underlying pathogenesis involves an aberrant immune response in which the immune system, originally intended to protect the host from external threats, mistakenly targets self-tissues. The pathogenesis of RA is orchestrated by a complex network of immune cells and soluble mediators, among which cytokines play a central role. The significance of cytokines in RA is evident from the high efficacy of cytokine-targeted therapies such as tumor necrosis factor (TNF)-α inhibitors and interleukin (IL)-6 inhibitors. In this review, we sought to divide the pathogenesis of autoantibody-positive RA into a pre-arthritis (preclinical) phase and an arthritis (clinical) phase, and discuss the roles of cytokines in terms of the ‘initiation of autoimmunity’, ‘development and persistence of synovitis’, and ‘progression of joint destruction’. In particular, we focus on cytokines that play central roles in RA pathogenesis across these stages, including IL-6 and TNF-α as major drivers of inflammation and joint damage, IL-1β and IL-17 as amplifiers of synovitis, IL-21 as a key mediator of T follicular helper cell (Tfh)/T peripheral helper cells (Tph)–B cell interactions, B cell-activating factor (BAFF) and A proliferation-inducing ligand (APRIL) as regulators of B cell survival and autoantibody production, and type I interferon (IFN) as a critical factor in the preclinical phase. To enhance the comprehensiveness of this narrative review, we conducted a literature search using PubMed. The keywords used included “rheumatoid arthritis,” “cytokines,” “autoimmunity,” “synovitis,” and “bone destruction.” We included both original and review articles published in English, mainly between January 2000 and June 2025. Articles were selected based on their relevance to the immunopathogenesis of RA, particularly those describing cytokine function, immune cell interactions, and tissue damage. Case reports and non-peer-reviewed sources were excluded.

## 2. Pre-Arthritis (Preclinical) Phase of RA

**Figure 1:**
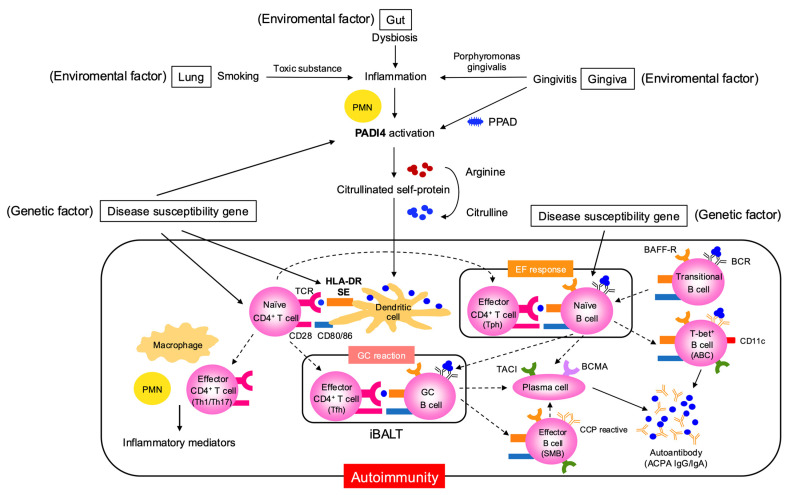
**Pathologic mechanisms linking environmental and genetic factors to autoimmunity in the preclinical phase of RA.** This figure details the interplay between genetic susceptibility (e.g., disease risk genes including PADI4) and environmental insults such as smoking, gingivitis caused by Porphyromonas gingivalis, and gut dysbiosis. It describes how these factors drive post-translational modification of self-proteins, notably citrullination catalyzed by PPAD and PADI4, creating neoantigens that breach immune tolerance. The process involves tissue sites such as the lung, gingiva, and gut, and depicts the initial activation of innate immune cells (e.g., neutrophils and dendritic cells), subsequent T and B cell activation through antigen presentation (via HLA-DR molecules and costimulatory signals such as CD28/CD80/86), and the fostering of local and systemic autoimmune responses and inflammation. ABC, age-associated B cell; ACPA, anti-citrullinated protein antibody; BAFF-R, B-cell-activating factor-receptor; BCMA, B-cell maturation antigen; EF, extrafollicular; GC, germinal center; iBALT, inducible bronchus-associated lymphoid tissue; PADI, peptidyl arginine deaminase; PPAD, porphyromonas gingivalis peptidylarginine deiminase; PMN polymorphonuclear neutrophil; TACI, transmembrane activator and CAML interactor; Tfh, T follicular helper; Tph, T peripheral helper.

The pre-arthritis phase of RA refers to the stage before the onset of clinical symptoms, during which autoimmunity has already begun, characterized by the appearance of autoantibodies such as anti-cyclic citrullinated peptide antibody (ACPA) and rheumatoid factor (RF) [1]. In this RA risk phase, genetic and environmental factors interact to drive disease progression (Figure 1). Genetic factors include susceptibility genes such as HLA-DR (Human Leukocyte Antigen-DR) shared-epitope (SE) alleles and the peptidyl arginine deiminase 4 (PADI4) gene, which encodes peptidyl arginine deiminase, involved in citrullination [2,3]. Environmental factors such as smoking, periodontitis and gut dysbiosis activate PADI4 in neutrophils, leading to the abundant production of citrullinated self-proteins [4,5,6]. These self-proteins are presented as citrullinated peptides by dendritic cell HLA-DR SE molecules, inducing the activation of naïve CD4^+^ T cells, thereby initiating autoimmunity [7]. Concurrently, the activation of naïve B cells reactive to these autoantigens is also induced [8]. In autoimmune diseases like RA, a breakdown of B cell tolerance results in an increased presence of autoreactive naïve B cells compared to healthy individuals [9].

**Figure 2:**
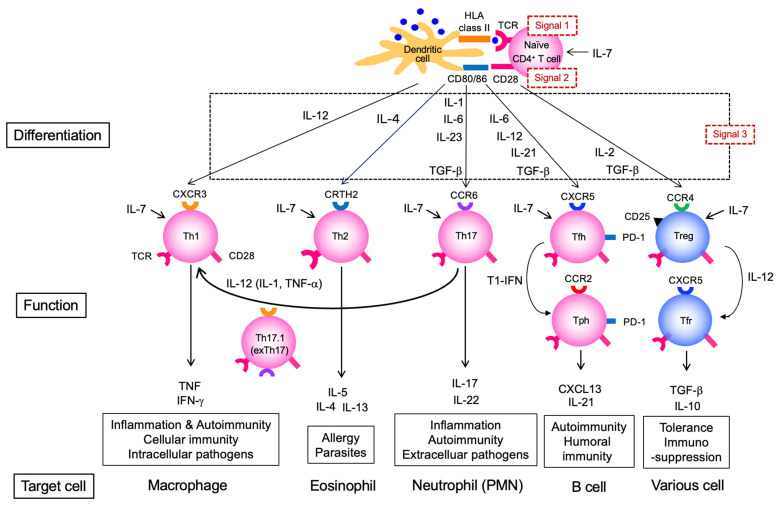
**Differentiation and functions of CD4^+^ T cell subsets and their roles in autoimmunity.** This figure outlines the differentiation pathways of naïve CD4^+^ T cells into effector subsets (Th1, Th17.1, Th2, Th17, Tfh, Tph, Treg, Tfr), driven by different cytokines and environmental cues. The specific functions and characteristic markers of each subset are diagrammed, including their involvement in cellular and humoral immunity, inflammation, immunosuppression, and tolerance. TCR, T cell receptor; Tfh, T follicular helper; Tfr, T follicular regulatory cell: Tph, T peripheral helper; Treg, regulatory T cell.

**Figure 3:**
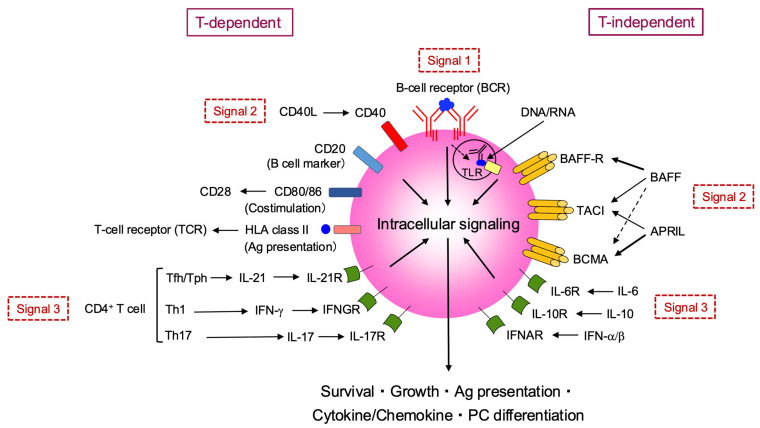
**B cell activation, differentiation, and effector functions in autoimmune responses.** This figure provides an overview of B cell activation via T-dependent and T-independent pathways. T-dependent activation requires three signals: antigen binding to the B-cell receptor (BCR) (Signal 1), co-stimulation via CD40-CD40L and CD28-CD80/86 interactions with CD4^+^ T cells (Signal 2), and cytokine signaling (Signal 3) from Tfh/Tph, Th1, or Th17 cells. T-independent activation also involves antigen/TLR binding (Signal 1) and additional signals (Signal 2) from BAFF/APRIL binding to BAFF-R, TACI, and BCMA, along with cytokine signaling (Signal 3) from IL-6, IL-10, and IFN-α/β. All these pathways converge on intracellular signaling to promote B cell survival, growth, and antigen presentation, cytokine/chemokine production, and plasma cell differentiation. BAFF, B-cell-activating factor; BCMA, B-cell maturation antigen; IFN, interferon; TACI, transmembrane activator and CAML interactor; PC, plasma cell.

Cytokines are critical for the functional maturation (differentiation into effectors) of both T and B cells. The activation of naïve CD4^+^ T cells requires antigen recognition via T cell receptor (TCR) (signal 1) and co-stimulation via CD28 (signal 2); however, differentiation into effector CD4^+^ T cells with specific cytokine-producing profiles requires additional cytokine stimulation (signal 3) (Figure 2). In local tissues, such as the lungs, differentiation into Th1 or Th17 subsets and the subsequent production of inflammatory mediators is observed, but a key feature in this stage is the production of autoantibody ACPA. This process critically involves Tfh and Tph subsets, which exhibit a strong B cell-helper capacity (Figure 2). Multiple cytokines are involved in Tfh differentiation, and in humans, as in Th1 cells, IL-12 plays an essential role; indeed, Tfh differentiation is impaired in individuals with IL-12 receptor β1 chain (IL12RB1) deficiency [10]. In addition to IL-12, cytokines such as transforming growth factor (TGF)-β are also important for human Tfh differentiation. Moreover, the marked reduction in circulating Tfh (cTfh) cells in patients with Signal Transducer and Activator of Transcription 3 (STAT3) deficiency suggests that the cytokines acting via the STAT3 pathway—such as IL-6 and IL-21—are also crucial [11]. Among the cytokines produced by Tfh cells, IL-21 is of central importance. B cells require cytokine stimulation to exert effector functions (Figure 3). IL-21 activates STAT3 in B cells and induces the transcription factor B lymphocyte-induced maturation protein 1 (BLIMP-1), which is critical for plasma cell differentiation [12].

As previously mentioned, RA patients harbor abundant autoreactive naïve B cells. These cells are influenced not only by T cell-derived IL-21 but also by other cytokines that disrupt their self-tolerance. Innate immune cells such as neutrophils, macrophages, and dendritic cells produce key B cell-modulating cytokines, including BAFF, APRIL, and IL-10 (Figure 3). BAFF promotes the differentiation, survival, and maturation of B cells [13]. Since naïve B cells express the BAFF receptor (BAFF-R), excessive BAFF availability may support the persistence of autoreactive B cells [14]. APRIL is critical for the long-term survival of plasma cells, which express its receptors the transmembrane activator and CAML interactor (TACI) and B-cell maturation antigens (BCMAs) [15,16]. Unlike BAFF and APRIL, IL-10 can also be produced by B cells themselves. It promotes plasma cell differentiation via STAT3 activation [17]. Interestingly, although APRIL facilitates the differentiation and survival of pathogenic (autoreactive) B cells, it has also been reported to promote IL-10-producing regulatory B cells (Bregs). Under APRIL stimulation, IgA^+^ Bregs are induced, which suppress T cell and macrophage-mediated inflammation through IL-10 and programmed death-ligand 1 (PD-L1) expression [18,19]. This duality of APRIL—supporting both pathogenic and regulatory B cell subsets—has important implications for therapeutic targeting and adverse event prediction in RA [20].

Tph cells were originally identified in RA joints as IL-21-producing PD-1^hi^ CD4^+^ T cells distinct from Tfh cells [21]. Notably, while Tfh cells express CXCR5, Tph cells express CCR2, indicating that Tfh cells are involved in B cell-help within lymphoid tissues, whereas Tph cells function in inflamed tissues such as synovial tissues in RA [22] (Figure 2). Interestingly, Tph cells themselves also produce CXCL13, a ligand for CXCR5, suggesting that they may recruit Tfh cells to inflammatory sites. Recent studies suggest that type I IFN can induce a shift from Tfh to Tph cells, a phenomenon that could potentially occur in inflammatory environments [23]. Tfh cells are involved in the germinal center (GC) reaction and promote somatic hypermutation (SHM) and class-switch recombination in GC B cells, mainly via IL-4 and IL-21 [24]. On the other hand, Tph cells induce the differentiation of naïve B cells into a distinct CD11c^+^ T-bet^+^ subset known as age-associated B cells (ABC) [25]. These T-bet^+^ B cells are frequently autoreactive [26], strongly implicating them in autoimmunity. In addition to IL-21, IFN-γ is crucial for their differentiation, as T-bet^+^ B cells are reduced when IFN-γ signaling is impaired [27]. IFN-γ signaling is also essential for the expression of CXCR3 on T-bet^+^ B cells, promoting their recruitment to inflammatory sites [28]. Interestingly, IFN-γ and IL-4 act antagonistically in driving T-bet^+^ B cell development, suggesting that the cytokine milieu may influence the choice between GC and extrafollicular pathways [29].

What, then, is the contribution of Tfh and Tph cells to pathogenesis during the preclinical phase? In RA lung lesions, structures resembling secondary lymphoid organs called inducible bronchus-associated lymphoid tissue (iBALT) are observed [30], suggesting that GC reactions mediated by Tfh cells may be occurring (Figure 1), though direct evidence is currently limited. ACPA is known to undergo variable domain glycosylation (VDG), which progressively increases even before the onset of arthritis and is already fully established by the time of clinical manifestation. Since this glycosylation is introduced through SHM [31], it again suggests the importance of GC reactions driven by Tfh cells during the preclinical phase. On the other hand, although much remains unclear regarding Tph cells, an increase in Tph cells has been observed in the peripheral blood of ACPA-positive individuals in the preclinical phase, suggesting their potential involvement in RA development [32,33]. Although this pertains to the clinical phase, Tph cells have also been reported in the sputum and lung tissues of RA patients [34]. Additionally, since elevated type I IFN are present in ACPA-positive individuals prior to RA onset [35], Tph differentiation may also occur during this phase. The presence of Tph cells implies possible co-localization with T-bet^+^ B cells. An analysis of bronchoalveolar lavage (BAL) fluid from ACPA-positive individuals in the preclinical phase revealed the presence of double-negative (IgD^−^ CD27^−^) B cells, possibly including T-bet^+^ B cells, in lung tissues, suggesting their involvement in autoantibody production [36]. These findings collectively support the notion that autoantibody production reflecting underlying autoimmunity begins during the preclinical phase.

Omics analyses in the preclinical phase of RA have provided evidence for the critical role of type I IFN. Notably, transcriptomic studies have demonstrated that ACPA-positive individuals and those with arthralgia prior to RA onset exhibit an type I IFN signature—defined as the upregulation of type I IFN-responsive genes—which is significantly associated with increased risk of progression to RA [37,38]. Individuals with high type I IFN signature expression had approximately twice the risk of developing RA, independent of the presence of autoantibodies such as ACPA and RF. This type I IFN signature also correlates with autoantibody titers and is implicated in immunological alterations such as immune cell activation and B cell proliferation. In addition, serum proteomic analyses conducted around the time of RA onset have revealed changes in molecular networks associated with IFN-α responses and lipid metabolism pathways [39]. Furthermore, proteomic analyses of sera from ACPA-positive individuals have shown that cytokines such as IL-1, IL-2, and IFN-γ correlate with ACPA variable-domain glycosylation (VDG), and cases that progressed to inflammatory arthritis exhibited strong activation of the JAK-STAT pathway [40].

To date, no studies have shown that the inhibition of specific cytokines during the preclinical phase can prevent the onset of RA. For instance, the administration of TNF-α inhibitors during the preclinical stage does not suppress disease development, likely because TNF-α plays a more prominent role during the arthritis (clinical) phase, as discussed later. In contrast, IL-6, which promotes the differentiation of Tfh and Tph cells as described above, may hold promise as a preventive target. Results from some studies suggest that IL-6 inhibitors and JAK inhibitors may have the potential to suppress disease progression in high-risk individuals—such as those who are autoantibody-positive or exhibit subclinical inflammation [41]. Notably, JAK inhibitors are of particular interest as a potential therapeutic strategy in the preclinical phase due to their capacity to regulate the activity of type I IFN, as described above. However, there is currently no evidence supporting its use for disease prevention, and further research is needed [42].

## 3. Arthritis (Clinical) Phase of RA

### 3.1. Autoimmunity

**Figure 4:**
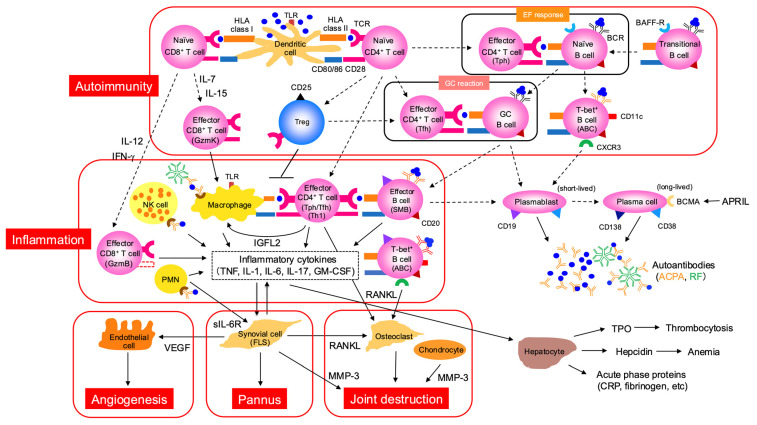
**Pathogenesis of clinical phase of RA is characterized by autoimmunity, synovitis, and joint destruction.** This figure delineates the intricate cellular and molecular events in autoimmunity, inflammation, pannus formation, and joint destruction. In the context of autoimmunity, dendritic cells activate naïve CD8^+^ T cells and naïve CD4^+^ T cells via HLA class I and HLA class II, respectively. These activated T cells can differentiate into effector CD8^+^ T cells (GzmK) and effector CD4^+^ T cells (Tfh). The EF response involves the interaction between effector CD4^+^ T cells (Tph) and naïve B cells, thereby generating T-bet^+^ B cells. The GC reaction involves effector CD4^+^ T cells (Tfh) and GC B cells. Ultimately, plasmablasts (short-lived) and plasma cells (long-lived), characterized by CD19, CD138, CD38, BCMA, and APRIL, produce autoantibodies such as ACPA and RF. Inflammation is driven by macrophages and NK cells, activated via TLR, leading to the stimulation of effector CD4^+^ T cells (Tph/Tfh/Th1) and effector B cells (SMB). PMNs and effector CD8+ T cells (GzmB) also contribute to the release of inflammatory cytokines like TNF, IL-1, IL-6, and IL-8. T-bet^+^ B cells are also implicated in the inflammatory process. These inflammatory processes contribute directly to pannus formation and subsequent joint destruction. Synovial cells (FLS), osteoclasts, and chondrocytes, through pathways involving RANKL and MMP-3, drive the development of pannus and eventual joint degradation. Additionally, inflammatory cytokines influence hepatocytes, leading to the production of acute phase proteins (CRP, fibrinogen, etc.) and hepcidin, which is associated with anemia. ABC, age-associated B cell; ACPA, anti-citrullinated protein antibody; APRIL, a proliferation-inducing ligand; BAFF, B-cell-activating factor; BCR, B cell receptor; BCMA, B-cell maturation antigen; FLS, fibroblast-like synoviocyte; Gzm, granzyme; IGFL2, insulin-like growth factor-like family member 2; MMP, matrix metalloproteinase; PMN, polymorphonuclear neutrophil; RANKL receptor activator of nuclear factor kappa B ligand; RF, rheumatoid factor; TPO, thrombopoietin; VEGF, vascular endothelial growth factor.

Even during the clinical phase of RA, T cells and B cells continue to mediate autoimmune responses within the joint. The RA synovium harbors diverse T cell subsets—particularly Tph cells—as well as B cells, which undergo clonal expansion, recognize self-antigens, produce cytokines, and promote plasma cell differentiation [4]. Comprehensive analyses comparing immune cell profiles between synovial tissue and peripheral blood in patients with early, treatment-naïve RA have shown that Tph cell-associated signatures serve as important biomarkers for treatment response and prognosis [43]. Circulating Tfh (cTfh) and Tph cells in RA patients exhibit distinct metabolic profiles: cTfh cells show heightened glucose metabolism, particularly glycolysis, and are more potent in B cell activation and antibody production, whereas Tph cells demonstrate elevated mitochondrial reactive oxygen species (mtROS) and express high levels of cytotoxicity-related molecules such as BLIMP-1 and T-bet, as well as markers of cellular senescence [44]. Moreover, synovial Tph cells show stronger recent TCR activation signatures and more pronounced clonal expansion compared to Tfh cells [45]. Interestingly, recent studies have identified two functionally and spatially distinct Tph subsets in RA: stem-like Tph (S-Tph) and effector Tph (E-Tph). S-Tph cells possess self-renewal and B cell-helping capacity within tertiary lymphoid structures (TLS), while E-Tph cells contribute to inflammation and tissue damage. Differentiation from S-Tph to E-Tph occurs within RA tissues [46]. Human Tph cells also produce a primate-specific secreted factor, insulin-like growth factor-like family member 2 (IGFL2), which correlates with RA disease activity [47]. IGFL2 functions as a cytokine that cooperates with TGF-β stimulation to promote CXCL13 production and induces the expression of numerous inflammatory and IFN-related genes (e.g., CXCL9/10/11) in monocytes and macrophages (Figure 4).

In patients with early RA who are DMARD-naïve, ABCs highly express chemokine receptors such as CXCR3 and adhesion molecules involved in homing to inflamed tissues, and represent a dominant B cell subset in synovial fluid [48]. Single-cell RNA sequencing analysis of ABCs in peripheral blood of RA patients revealed that the proportion of ABCs correlates with disease activity and serum TNF-α levels, and identified spleen tyrosine kinase (Syk) as a key regulator of the myeloid-like phenotype of ABCs [49]. The aberrant expansion of ABCs is implicated in RA pathogenesis and represents a potential therapeutic target [50].

As described earlier, IL-6 plays a critical role in the differentiation of both Tfh and Tph cells; in addition, IL-6 directly acts on B cells, promoting plasma cell differentiation via STAT3 activation (Figure 3 and Figure 4). In this phase, members of the TNF cytokine family, BAFF and APRIL, are also upregulated in RA serum and synovial tissue (Figure 4) [14]. However, ACPA titers show only weak correlation with RA disease activity and treatment response, and generally remain stable over time. This may be partly explained by the fact that ACPA-producing B cells have already acquired immunological memory—as evidenced by VDG—before the onset of arthritis [51]. Moreover, as ACPA-producing cells, CXCR3^+^ switched memory B cells (SMB), plasmablasts, and long-lived plasma cells residing in the bone marrow have been implicated, rather than the aforementioned T-bet^+^ B cells (Figure 4) [52,53]. In contrast, rheumatoid factor (RF), which is typically an IgM autoantibody targeting the Fc region of IgG, appears to be produced through mechanisms that involve innate immune signaling, unlike ACPA [54].

### 3.2. Development and Maintenance of Synovitis

In addition to autoimmunity, the clinical phase is marked by synovial inflammation. At this stage, not only T and B cells, but also innate immune cells such as macrophages and fibroblast-like synoviocytes (FLS), play key roles. Synovial macrophages play a central role in promoting chronic synovitis and joint destruction by producing pro-inflammatory cytokines—particularly TNF-α, IL-1β, and IL-6 (Figure 4). Synovial macrophages are classified into M1 (pro-inflammatory) and M2 (anti-inflammatory) phenotypes; in RA, there is a predominance of M1 macrophages, which are characterized by the elevated production of TNF-α and IL-1β [55]. Among the cytokines, IFN-γ, secreted by Th1 cells, potently drives M1 polarization, while granulocyte–macrophage colony-stimulating factor (GM-CSF), which is highly expressed in the synovium, is also critical for maintaining the M1 phenotype in monocyte-derived macrophages. TNF-α and IL-1β secreted by M1 macrophages further amplify the inflammatory loop [56,57,58].

FLS, when stimulated by cytokines such as TNF-α, IL-1β, IL-6, and TGF-β, increase the expression of adhesion molecules (e.g., ICAM-1) and enhance crosstalk with immune cells—particularly CD4^+^ T cells and macrophages—leading to elevated production of inflammatory cytokines and chemokines [59]. Under TNF-α stimulation, FLS particularly promote direct cellular interactions with T cells and enhance their activation and differentiation. Moreover, FLS contribute to bone destruction through the production of receptor activator of nuclear factor κB ligand (RANKL), and a subset of ITGA5^+^ FLS expressing TGF-β, periostin (POSTN), and CCL5 has been implicated in promoting Tph differentiation and forming inflammatory niches [60]. Interactions between FLS and macrophages further increase the production of inflammatory cytokines and matrix metalloproteinases (MMP2 and MMP9), exacerbating chronic inflammation and tissue damage. Recent single-cell and spatial transcriptomic analyses have revealed the functional heterogeneity of FLS subsets, highlighting their emerging importance as therapeutic targets in RA [61].

In addition to Th1 cells, Th17 cells produce IL-17A and other cytokines that stimulate FLS and macrophages, thereby amplifying inflammation. Under inflammatory conditions (e.g., in the presence of IL-12, IL-1 or TNF-α), Th17 cells may transdifferentiate into Th17.1 (or exTh17) cells, which co-produce IFN-γ (Figure 2). These Th17.1 cells are increased in RA synovial fluid and have been reported to be more pro-inflammatory and treatment-resistant than classical Th17 cells [62].

In early RA, synovial T cells exhibit a Th1-skewed profile, with high production of inflammatory cytokines such as IFN-γ, while IL-4 and Th2 cells are relatively scarce. Nonetheless, IL-4 production is detectable in the synovium of early RA and may contribute to both anti-inflammatory effects and enhanced antibody production through B cell activation. IL-4 promotes B cell differentiation and antibody production—particularly IgE and autoantibodies—and may be involved in the autoimmune processes of RA. It also modulates inflammatory cytokine secretion from synovial fibroblasts and neutrophils [63]. In chronic or advanced RA, however, a Th1/Th17-dominant milieu prevails, and synovial T cells become resistant to Th2 polarization, thereby limiting IL-4’s anti-inflammatory function. Nevertheless, IL-4 can still suppress neutrophil infiltration into the joints and enhance FcγR2b expression, contributing to inflammation control [64,65]. As previously noted, Tph cells play a central role in RA synovium, but Tfh cells are also present. Tfh cells can be subdivided into Tfh1, Tfh2, and Tfh17 subsets based on their cytokine profiles. During the clinical phase, Tfh2 cells—which produce IL-21 and IL-4—are considered more functionally relevant than Tfh1 cells, which produce IL-21 and IFN-γ. Tfh2 cells strongly contribute to B cell activation and autoantibody production [66]. Moreover, in peripheral blood of RA patients, increases in Tfh2 and Tfh17 (producing IL-21 and IL-17) subsets have been observed, whereas Tfh1 cells appear to have lower B cell-helper activity.

In contrast to pathogenic subsets, regulatory CD4^+^ T cell subsets also exist (Figure 2). T regulatory (Treg) cells are induced by IL-2 and TGF-β and secrete immunosuppressive cytokines such as IL-10 and TGF-β. Tregs maintain immune homeostasis by suppressing effector T cell responses. In co-culture experiments with human conventional CD4^+^ T cells (Tconv), Tregs have been shown to strongly suppress both the proliferation and cytokine production of Tconv cells. This suppression is primarily contact-dependent, as the effect is abolished when Tregs and Tconv cells are separated by a transwell, indicating that direct cell–cell interactions, in addition to soluble factors, are essential [67]. Moreover, the suppressive capacity of Tregs varies by subset. Memory-type Tregs (CD45RA^−^ FoxP3^hi^) potently inhibit early Tconv activation—such as activation marker expression and cytokine secretion—whereas their ability to suppress proliferation is comparable to that of naive Tregs [68]. Tregs that express follicular-homing molecules such as CXCR5, migrate into follicles, and acquire suppressive functions are referred to as T follicular regulatory (Tfr) cells. Recent human studies have shown that low-dose IL-12 induces Tfr differentiation programs in activated Tregs by activating STAT4 and upregulating Tfr-associated genes (Figure 2) [69]. In untreated early RA patients, Treg cells are significantly reduced, showing negative correlations with disease activity and autoantibody titers [70]. Additionally, peripheral Tfr cells are decreased in active RA compared to remission [71]. These findings suggest reductions in both Treg and Tfr populations during the clinical phase of RA (Figure 4). In humans, impaired Treg function and numerical deficiency contribute to the breakdown of peripheral B cell tolerance checkpoints, leading to the accumulation of autoreactive B cells and subsequent autoantibody production [72]. Interestingly, TNF inhibitors restore Treg populations in RA [73], and IL-6 inhibitors have been shown to restore both Treg and Tfr cells [74,75]. Meanwhile, T-bet^+^ B cells, which express CXCR3, are abundant in the joint and contribute to the inflammatory milieu by producing TNF-α and IL-6 [76] (Figure 4).

IL-8 produced by macrophages promotes the migration and activation of neutrophils, playing a critical role in sustaining chronic inflammation in RA [77]. Recent single-cell analyses have demonstrated the expansion of IL1B^+^ pro-inflammatory macrophages in the RA synovium [78]. FLS also contribute to IL-1β production. In addition to these cytokines, TNF-α and IL-6 are especially important in the inflamed joint microenvironment. TNF-α acts on various synovial cell types, with FLS being particularly important targets. TNF-α stimulates FLS to produce inflammatory cytokines and chemokines, promotes their proliferation and migration, enhances resistance to apoptosis, and collectively drives chronic synovitis [79].

IL-6, on the other hand, plays a central role in amplifying inflammation and inducing tissue damage in the joint. IL-6 signaling proceeds via two primary pathways: classic signaling and trans-signaling. In classic signaling, IL-6 acts on cells that express membrane-bound IL-6 receptor (mIL-6R), such as neutrophils, macrophages, and hepatocytes. In trans-signaling, IL-6 forms a complex with soluble IL-6R (sIL-6R), allowing it to act on nearly all cells that express the signal transducer gp130. This pathway leads to broader and sustained inflammation and is considered more relevant to RA pathogenesis [80]. In RA, concentrations of IL-6 and sIL-6R are elevated in the joint, enabling IL-6 to transmit inflammatory signals to cells such as FLS and chondrocytes that lack mIL-6R. Current IL-6 inhibitors target IL-6R and therefore block both classic and trans-signaling. Neutrophils are considered a major source of sIL-6R in the joint, generated via shedding of mIL-6R (Figure 4).

What, then, are the primary sources of IL-6 in the joint? Recent single-cell studies have identified THY1 (CD90)^+^ sublining FLS as the main producers of IL-6 [78]. These findings underscore the central role of FLS in driving inflammation in the clinical phase of RA. Beyond local effects, IL-6 also contributes to systemic inflammation by inducing the hepatic production of hepcidin and thrombopoietin (TPO), leading to anemia of chronic disease and thrombocytosis. IL-6 further stimulates the acute-phase response, promoting the synthesis of C-reactive protein (CRP), fibrinogen, and other proteins (Figure 4).

Omics technologies, including transcriptomics and single-cell RNA sequencing, have been widely employed to elucidate RA pathogenesis. These analyses have revealed that multiple cytokines—such as TNF-α, IL-6, IL-17, IL-1β, and GM-CSF—play central roles in inflammation and tissue destruction in RA. The cytokine profiling of blood from RA patients has also demonstrated correlations between disease activity and levels of cytokines such as IL-17A and TNF-α [4,81]. Furthermore, plasma proteomics comparing RA patients and healthy controls has uncovered abnormalities in molecular networks centered around immune regulation, intracellular signaling, hematopoiesis, and cytokine modulation, including STAT1, TNF, and CD40. In addition, altered levels of osteocalcin (involved in bone metabolism), apolipoprotein A-I, and metallothionein-2 (an antioxidant protein) were also observed [82]. Proteomic analyses of neutrophils in RA synovial tissue have revealed a highly activated state, characterized by the massive release of myeloperoxidase (MPO) and ROS, which are major contributors to local protein carbamylation. Since carbamylated proteins can become targets of autoantibodies, they may contribute to the progression of RA [83].

Although the focus thus far has been on CD4^+^ T cells, CD8^+^ T cells also play important roles in RA pathogenesis (Figure 4). Recent single-cell studies have revealed expansions of cytotoxic CD8^+^ T cells, particularly granzyme B (GzmB)^+^ cells, in the peripheral blood and synovial tissue of ACPA-positive RA patients. These cells are activated by citrullinated antigens and contribute to synovitis and tissue destruction [84]. GzmB degrades components of the synovial extracellular matrix—such as tenascin-C—thereby generating pro-inflammatory fragments [85]. In contrast, a distinct subset of GzmK^+^ CD8^+^ T cells have recently gained attention. These cells are abundant in the sublining regions of RA synovium and exhibit limited cytotoxicity, but maintain chronic inflammation by producing inflammatory cytokines such as IFN-γ, activating complementary cells, and interacting with stromal cells [86]. The differentiation of these two CD8^+^ T cell subsets is also distinct (Figure 4): GzmK^+^ cells predominantly display central memory (CM) or effector memory (EM) phenotypes and respond strongly to cytokines such as IL-7 and IL-15. Under TCR stimulation, they downregulate GzmK and upregulate GzmB [87]. In contrast, GzmB^+^ cells represent more terminally differentiated effector phenotypes and are primarily induced via TCR stimulation, with IL-12 and IFN-γ acting as key cytokines [88]. We have also identified a subset of CD8^+^ T cells with B cell-helper capacity. These cells produce cytokines such as IFN-γ and IL-21 and share functional characteristics with Tph cells. They are abundant in the joints of autoantibody-positive RA patients [89]. Intriguingly, age-associated CD4^+^ T cells (ThA) were recently reported to possess a dual functionality, exhibiting both cytotoxic properties like CD8^+^ T cells and B cell-helper functions, supporting autoantibody production [90].

In addition to CD8^+^ T cells, NK cells also play important roles within RA joints. CD16^+^ NK cells are increased in RA synovial tissue and are activated through immune complexes to produce IFN-γ. This NK-derived IFN-γ promotes the expansion of CD90^+^ FLS, which express high levels of HLA-DR and inflammatory cytokines such as IL-6. These activated FLS enhance CD4^+^ T cell activation via antigen presentation and contribute to the maintenance of chronic inflammation and autoimmunity in RA [91].

Angiogenesis in RA synovium is promoted by various factors, including vascular endothelial growth factor (VEGF), TNF-α, IL-6, and IL-17. Among these, VEGF is considered the principal pro-angiogenic factor in RA [92]. FLS constitutively express VEGF, and their expression is further enhanced by hypoxia and pro-inflammatory cytokines [93]. TNF and IL-6 inhibitors, such as infliximab and tocilizumab, suppress VEGF production and are positively correlated with reductions in synovitis severity, as assessed by ultrasound [92].

### 3.3. Progression of Joint Destruction

During the clinical phase of RA, joint destruction progresses and ultimately leads to irreversible deformity and functional impairment. TNF-α and IL-1β induce FLS and chondrocytes to express MMP-3, which contributes to cartilage matrix degradation [94]. Furthermore, IL-6–mediated trans-signaling promotes RANKL expression by FLS, thereby inducing osteoclast differentiation and bone resorption [4].

IFN-γ, in addition to modulating FLS function, enhances their motility and invasiveness, contributing to the destructive remodeling of joint tissues. This process is mediated via the JAK2–FAK signaling pathway and is suppressed by JAK inhibitors [95]. Osteoclasts are central to bone erosion. TNF-α, IL-6, IL-1β, and IL-17 upregulate RANKL expression in FLS, promoting osteoclastogenesis and activation. IL-1β enhances the bone-resorbing activity of osteoclasts and also contributes to RANKL-independent activation [96]. Moreover, TNF-α, in the presence of M-CSF, can induce osteoclast differentiation from murine macrophages independently of RANKL [97]; however, in human monocytes, synergistic actions with other cytokines—particularly IL-6 and TGF-β—are considered essential [98,99]. Since FLS do not express mIL-6R, trans-signaling is again critical for IL-6-induced RANKL expression in these cells [100]. Transcription factors such as Ets2 and SOX5 are also activated under IL-6 trans-signaling and contribute to RANKL upregulation [101].

Although FLS are the primary source of RANKL in RA, we showed that double-negative B cells—including T-bet^+^ subsets—and switched memory B cells also express RANKL [102]. In untreated RA patients, the frequency of T-bet^+^ B cells correlates with bone erosions, joint space narrowing, and modified total sharp score (mTSS) [103]. In addition, T cells can also serve as a source of RANKL in RA (Figure 4). CD4^+^ T cells from RA patients have been shown to express RANKL upon stimulation with inflammatory cytokines such as IL-21 and IL-23, thereby contributing to bone destruction [104]. However, recent molecular analyses have demonstrated that the majority of RANKL-producing cells in RA synovium are FLS [105]. Interestingly, the inhibition of RANKL alone effectively suppresses bone erosion but does not sufficiently control synovitis, suggesting that the mechanisms of joint inflammation and bone destruction are at least partially distinct [106].

## 4. Conclusions

RA represents a paradigm of chronic inflammation in which cytokine networks orchestrate the continuum from autoimmunity to synovitis and joint destruction. Our synthesis of current evidence highlights that although TNF-α and IL-6 remain the most dominant and therapeutically validated cytokines, a broader spectrum—including IL-1β, IL-17, BAFF/APRIL, type I IFN, and novel mediators identified by single-cell and proteomic technologies—collectively shape disease progression. This complexity underscores why blockade of a single cytokine may fail in a subset of patients and points to the need for strategies that can modulate multiple signaling pathways simultaneously, as exemplified by JAK inhibitors.

From the authors’ perspective, one of the most pressing frontiers is the preclinical phase. Cytokine perturbations clearly precede the onset of arthritis, yet reliable biomarkers that predict transition to overt disease remain elusive, and preventive interventions have not been firmly established. Deeper exploration of this “window of opportunity,” through longitudinal profiling and molecular stratification, may ultimately allow us to intercept disease before irreversible tissue injury occurs. At the same time, heterogeneity within the synovium—such as FLS subsets driving fibrosis, or pathogenic Tph/T-bet^+^ B cell interactions sustaining chronicity—reminds us that RA is not a uniform condition. Future therapies will likely need to account for such endotype-specific differences.

In conclusion, while cytokine-targeted therapies have already transformed RA management, the continued integration of omics technologies, spatial immunology, and clinical observation holds the promise of a more precise and preventive approach. We envision a future in which cytokine signatures not only guide treatment selection but also enable early intervention strategies, redefining the trajectory of RA from inevitable progression to a potentially preventable disease.
